# The Mechanism of Quality Changes in Grass Carp (*Ctenopharyngodon idella*) During Vacuum-Assisted Salting Brining with Physicochemical and Microstructural View

**DOI:** 10.3390/foods14040657

**Published:** 2025-02-15

**Authors:** Jianrong Ma, Jian Shi, Ruiying Lv, Xin Jiang, Qingqing Jiang, Dajun Wang, Shumin Zhang, Wenzheng Shi

**Affiliations:** 1 College of Food Science and Technology, Shanghai Ocean University, Shanghai 201306, China; mjr1207@163.com (J.M.); s19895905220@163.com (J.S.); 15837028985@163.com (R.L.); 13053523375@163.com (X.J.); qqjiang@shou.edu.cn (Q.J.); wangdajun@haihofoods.com (D.W.); zhangmin0530@163.com (S.Z.); 2 Marine Biomedical Science and Technology Innovation Platform of Lin-Gang Special Area, Shanghai 201306, China

**Keywords:** vacuum impregnation, brining efficiency, water-holding capacity, microstructure, grass carp

## Abstract

This study investigated the effects of vacuum impregnation (V) and atmospheric pressure impregnation (C) on the quality and microstructure of grass carp (*Ctenopharyngodon idella*) fillets during a 120 min brining period under 5.8% NaCl (*w*/*v*) and 4 °C. Vacuum impregnation significantly enhanced brining efficiency, achieving higher salt content (2.01%) and accelerated mass transfer kinetics, as evidenced by Peleg model parameters. Both treatments improved the water-holding capacity (WHC), increasing from 80.16% to 91.47% (C) and 89.92% (V), while reducing free water proportion. A microstructural analysis revealed a reduction in extracellular space in fillets, which further affected the fillet WHC and texture. Vacuum brining also mitigated lipid oxidation, yielding lower thiobarbituric acid-reactive substances (TBARSs: 0.237 mg MDA/kg). Texture analysis indicated reduced hardness and increased springiness/cohesiveness in fillets. Despite a slight decrease in lightness, vacuum-assisted brining preserved sensory quality and aligned with low-salt dietary trends. These findings underscore vacuum technology’s potential to optimize brining efficiency and product quality in industrial applications.

## 1. Introduction

Grass carp (*Ctenopharyngodon idella*) is a significant farmed fish species in China which is favoured for its health benefits, rapid growth, and its white, soft flesh with rich nutritional value [1]. However, grass carp fillets are high in water content and rich in endogenous enzymes, which can lead to several quality issues during processing and transportation. Specifically, the high water content in grass carp fillets can make them more susceptible to softening, as the moisture can facilitate enzymatic and microbial activities that break down the muscle structure. Additionally, the rich presence of endogenous enzymes, such as proteases and lipases, can cause discoloration by degrading proteins and lipids, leading to the formation of undesirable compounds [2].

Salting brining is a pivotal processing method for fishery products, effectively extending shelf life by inhibiting microbial growth, enhancing flavour, and improving texture through controlled dehydration and protein denaturation [3,4]. Additionally, salting contributes to the unique sensory characteristics of preserved fish products, making it indispensable in traditional aquatic food processing. Traditionally processed fish are high in NaCl in brined meat products to achieve the desired quality requirements, but this can lead to excessive sodium intake by consumers [5]. A high dietary sodium intake may lead to hypertension, cardiovascular disease, and diabetes mellitus [6,7].

In wet brining processes, the concentration of sodium chloride (NaCl) critically governs the structural and physicochemical transformations of muscle tissues, particularly in fish. At low salt concentrations (<3%), myofibrillar proteins tend to hydrate, leading to fibre swelling and an increased water-holding capacity. Conversely, higher salt concentrations (>8%) induce myofibrillar protein dissolution and fibre contraction, promoting excessive water loss and textural hardening [8,9]. These opposing effects create a dynamic equilibrium in intermediate salt concentrations, where moderate osmotic pressure balances hydration and dehydration, optimizing mass transfer efficiency while preserving tissue integrity. Preliminary studies suggest that 5.8% NaCl (≈1 mol/L) induces moderate fibre swelling, enhancing brine absorption without irreversible structural damage [10].

Vacuum technology has gained prominence in regard to overcoming the inherent limitations of traditional wet brining, particularly in terms of efficiency and mass transfer dynamics. Conventional brining relies on passive diffusion driven by osmotic gradients, a slow process that often necessitates prolonged processing times or high salt concentrations to achieve the desired effect. In contrast, vacuum-assisted brining exploits reduced pressure to create a synergistic driving force that enhances solute penetration [11,12,13]. By lowering the external pressure, intracellular air and interstitial fluids are rapidly evacuated from the muscle matrix, collapsing structural barriers and accelerating the diffusion of salt ions into the tissue [14].

Despite these advantages, systematic comparisons of quality evolution between vacuum-assisted and traditional static brining remain limited, particularly in aquatic products. This study aims to elucidate the dynamic changes in physicochemical properties and microstructural alterations in grass carp fillets during a 120 min brining process under vacuum-assisted and atmospheric conditions. Additionally, the corresponding brining kinetics will be quantified to evaluate the efficiency of vacuum technology in accelerating mass transfers at reduced salt concentrations. The findings will provide theoretical insights into optimizing low-salt brining processes while ensuring product quality, aligning with global trends toward healthier processed foods.

## 2. Materials and Methods

### 2.1. Preparation of Samples

A total of eight fresh grass carp (weight 3500 ± 500 g, total length 56.5 ± 6.7 cm) were transported alive to the laboratory in Shanghai, China, with an oxygen supply system. Upon arrival, the fish were humanely stunned using a rubber mallet to facilitate euthanasia. Following this, the head and viscera were removed. The fish were then dissected along the spinal column to extract the dorsal muscle sections. From these muscles, samples measuring 2.0 cm × 2.0 cm × 1 cm were prepared, aligning with the muscle tissue’s grain.

The prepared fillets were randomly assigned to two experimental groups for brining, namely vacuum impregnation (V) and conventional no-vacuum impregnation at atmospheric pressure (C), with 60 fillets in each group, respectively. Fillets were submerged in a brining solution consisting of a 5.8% *w*/*w* NaCl solution, brining a fish to a fluid weight ratio of 3:1, conducted at 4 °C. For the vacuum impregnation group, fillets were treated using a UCHINO model HU-HYZZ20-01 vacuum marinade machine set to a vacuum level of −20 kPa. The fillets were removed at specified time intervals (15, 30, 45, 60, 90 and 120 min) during the brining process, patted dry to remove surface water, and then individually packed for further analysis.

Notably, 10 fresh grass carp fillets were taken for the unsalted group, labelled 0 min, and included as a baseline for comparison with the salted groups to assess the impact of the brining process.

### 2.2. Determination of Salt Content

Salt concentration in the fish samples was determined using a PAL-ES1 salinometer (Atago Co., Ltd., Tokyo, Japan). The procedure involved taking 1.00 g of the fish sample (*W*_0_) and combining it with 10 mL of distilled water in a homogenization container. This mixture was then thoroughly homogenized to ensure even distribution of the salt throughout the solution. After homogenization, the mixture was filtered to remove any solid particles, and the filtrate was used for a salt content analysis.

The NaCl content (*X*_1_) in the filtrate was measured using the salinometer, and the salt content in the fish sample was calculated using the following equation:

(1) $$NaCl\;content\left(\%\right)=X_{1}\times \left(W_{0}+10\right)$$

This equation accounts for the initial weight of the sample and the added distilled water, providing an accurate determination of the salt content in the fish fillets.

### 2.3. Modelling Mass Transfer Kinetics During Brining

The mass transfer kinetics during brining were modelled using Peleg’s two-parameter non-exponential empirical model [15]. This model is capable of predicting the mass transfer kinetics over extended periods based on experimental data obtained in a relatively short timeframe. The model equation is as follows:

(2) $$\begin{array}{c} X_{t}^{NaCl}\;=\;X_{0}^{NaCl}\;+\;\left(\frac{t}{k_{1}+t*k_{2}}\right) \end{array}$$

where $X_{t}^{NaCl}$ was the model-predicted sample salt content at moment *t*; $X_{0}^{NaCl}$ was the fresh sample salt content. The Peleg constant k_1_ was related to the initial mass transfer rate, and the Peleg constant k_2_ was related to the salt content at t →∞.

The experimental data for NaCl content in the vacuum impregnation (V) and atmospheric pressure brining groups (C) were fitted as a function of brining time using this model. The Peleg model was fitted to the experimental data using the nonlinear fitting operation in Origin 2021 software. To assess the adequacy of the model for the experimental data, regression coefficients (R^2^) and the root mean square error (RMSE) were calculated [16,17]. The RMSE quantifies the difference between the predicted and experimental values of salt content and is given by:

(3) $$RMSE=\frac{1}{N}\sqrt{\sum_{i=1}^{N}{\left(C_{pred}-C_{exp}\right)}^{2}}$$

where $C_{pred}$ and $C_{exp}$ are the predicted and experimental parameters and *N* is the number of experimental values.

### 2.4. Determination of the WG (Water Gain)

Fish water content was determined according to the AOAC method [18], and the WG was calculated by Equation (4):

(4) $$∆WG\left(\%\right)=\frac{m_{t}\times X_{t}^{w}-m_{0}\times X_{0}^{w}}{m_{0}}$$

where $X_{t}^{w}$ and $X_{0}^{w}$ were the water content of the brine t-moment and the fresh sample, respectively; *m*_t_ and *m*_0_ were the weight of the fresh sample at the brine t-moment and before brining, respectively.

### 2.5. Determination of the Content of Released Proteins During Brining

The protein content released during salting out was determined according to Huang [10]. Bovine serum protein was used as a standard protein solution to determine the protein quantification in the NaCl solution by the bicinchoninic acid assay.

(5) $$Content\;of\;released\;protein\left(\%\right)=\frac{C\times V}{m_{0}}\times 100$$

where *m*_0_ was the weight of the fish fillets before salting (g); *V* was the volume of the leftover NaCl solutions (mL); and *C* was the protein content in NaCl solutions (g/mL).

### 2.6. Determination of Salting Yield

The weight of the fish before salting (*W*_1_) and after salting (*W*_2_) was recorded, and the salting yield (*Y*) was calculated according to Equation (6):

(6) $$Y\left(\%\right)=\frac{W_{1}}{W_{2}}\times 100$$

### 2.7. Observation of Tissue Microstructure

The effect of salting conditions on the microstructure of grass fish fillets was assessed using light microscopy (LM), in accordance with Jiang [19]. For the LM analysis, samples were initially fixed in a solution comprising 75% absolute ethanol and 25% acetic acid (*v*/*v*) for 24 h at 4 °C. Subsequently, they were rinsed three times using deionized water and trimmed into blocks measuring 5.0 mm × 5.0 mm × 3.0 mm. Dehydration of the samples was carried out through a graded ethanol series from 50% to 100%. Following tissue fixation, dehydration, paraffin embedding, and sectioning, the samples were observed under a light microscope MS500W (Shanghai Metz Precision Instruments Co., Ltd., Shanghai, China).

### 2.8. Determination of Transverse Relaxation Time (T_2_)

The relaxation times using low-field 1 H NMR were evaluated for sample portions placed in cylindrical tubes (60 mm in diameter). The measurements of the transverse relaxation time (*T*_2_) were performed on a MesoMR23-060H-I pulse NMR analyzer (Shanghai Niumai Electronic Technology Co., Shanghai, China). The *T*_2_ was measured at 25 °C using the Carr–Purcell–Meiboom–Gill (CPMG) pulse sequence with 8 scans, a sampling frequency of 200 kHz, and 3.5 s between scans. The 90° pulse width was 17.00 μs, and the 180° pulse width was 37.05 μs.

### 2.9. pH Determination

An amount of 1g of fish meat was added with pre-cooled distilled water and homogenized at 1000 r/min for 1 min, then centrifuged at 10,000 r/min for 10 min. After filtration, the filtrate pH was measured using a pH metre (FE20, Mettler Toledo Co., Shanghai, China).

### 2.10. Determination of Cooking Loss and Water-Holding Capacity

Fish fillets were accurately weighed and dried at 105 °C until constant weight to determine the water content. The samples were packed in cooking bags and steamed in a water bath at 100 °C for 5 min. After cooling to 4 °C, the cubes were wiped with filter paper to remove the surface juices and weighed; the weight before steaming was recorded as *M*_1_ and the weight after steaming was recorded as *M*_2_. Cooking loss was calculated using Equation (**7**), as follows:

(7) $$Cooking\;loss\left(\%\right)=\frac{M_{1}-M_{2}}{M_{1}}\times 100$$

The formula for determination of water-holding capacity is as follows:

(8) $$WHC\left(\%\right)=\frac{X_{2}\times M_{2}}{X_{1}\times M_{1}}\times 100$$

where *X*_1_ and *X*_2_ represent the water content (%) before and after cooking, respectively.

### 2.11. Determination of Colour Characteristics

The colour characteristics of the fillets were measured using a colorimeter (CR-40, Konica Minolta Sensing Inc., Tokyo, Japan) to determine L*, a*, and b*.

### 2.12. Measurement of Thiobarbituric Acid-Reactive Substance (TBARS) Values

TBA was determined according to the method of Jiang et al. The fish sample (1.00 g) was added to 10 mL of 7.5% TCA solution containing 0.1% EDTA and homogenized at 1500 r/min for 1 min. After filtration, the mixture of TBA solution (0.02 mol/L) and filtrate was incubated in boiling water for 40 min and then cooled to room temperature. The TBARS value in fish was calculated from the absorbance at 532 nm with reference to the 1,1,3,3-tetraethoxypropane standard curve. The results of TBARS values were expressed as mg MDA/kg.

### 2.13. Determination of Textural Properties

The hardness, springiness, and cohesiveness of fish fillets were measured using a texture analyzer (TA. XT plus, Stable Micro System, Godalming, UK). The prepared fillets in the size of 2.0 cm × 2.0 cm× 1.0 cm were compressed twice using a P/6 cylindrical probe. The trigger force was 5 g, the test rate was 1.0 mm/s, the maximum compression ratio was 20%, and the dwell time between the compressions was 5 s. The peak force of the first compression cycle represented hardness, springiness was the high degree of recovery after the first compression, and cohesiveness was a measure of internal bonding and resistance to chewing food.

### 2.14. Statistical Analyses

The results of the time factor were analyzed by one-way ANOVA using SPSS 23.0 and multiple comparisons of means using Duncan’s method (*p* < 0.05); the significance between the two groups was analyzed using an independent samples *t*-test (*p* < 0.05). Data were expressed as mean ± standard deviation and plotted using Origin Pro 2024b.

## 3. Results and Discussion

### 3.1. Changes in Salt Content of Grass Carp Fillets During the Brining

**Figure 1:**
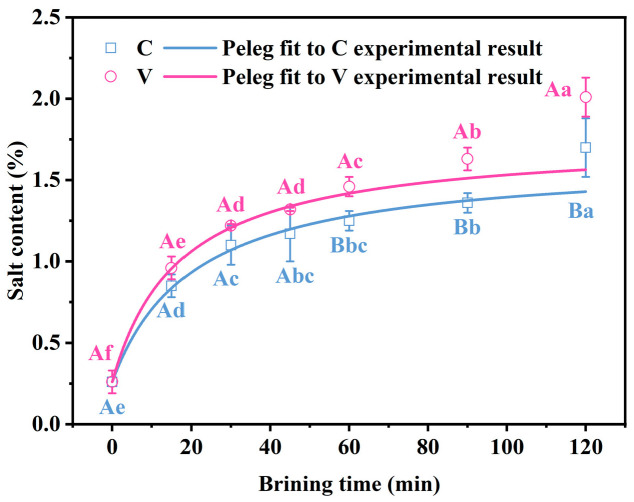
Changes in the salt content of grass carp fillets during the brining. The curves are the results of the Peleg fitting control experiments. V denotes vacuum impregnation and C denotes conventional non-vacuum impregnation at atmospheric pressure. Note: Different capital letters indicate significant differences (*p <* 0.05) between the two treatments; different lowercase letters indicate significant differences (*p <* 0.05) between different brining periods (same below).

The changes in salt content during the salting process of grass carp fillets are illustrated in Figure 1. For both experimental groups, the salt content significantly increased (*p* < 0.05) with the extension of salting time. After 45 min of salting, the salt content in the V group was notably higher than that in the C group (*p* < 0.05), with the rate of increase subsequently slowing down for both groups. This initial rapid increase in salt content is likely attributed to the substantial concentration gradient between the brine and the surface of the grass carp at the commencement of the process. As salting time progresses, the osmotic pressure inside the fillet with the solution decreases, leading to a decelerated rate of salt uptake. By the end of the brining process, the salt content rose to 2.01% and 1.70% for the V and C groups, respectively. Thus, a 100 g serving of fish products contributes about 33.44% to the daily sodium intake under conventional processing methods, while the contribution under a vacuum-assisted salting technique is about 39.54%. This product can be considered healthy. Such levels are considered healthy and align with the preferences for lightly salted products in Southern Europe, where such products have gained popularity [20]. Brining for 120 min resulted in light salting of grass carp fillets for both treatments [21], but the vacuum impregnation was more efficient.

The Peleg model was employed to predict the mass transfer kinetics from the experimental data. The results were nonlinearly fitted, and the fitted curves are presented in Figure 1. Table 1 presents model constants; the R^2^ of the samples from the two treatments were 0.9827 and 0.9245, respectively, and the RSEM were 0.0397 and 0.0658. These values indicate a good fit of the experimental data to the model. The rate constant k1, associated with the initial mass transfer rate, and the rate constant k_2_, related to the equilibrium salt concentration, were identified. The rate constant k_1_ is related to the mass transfer rate at the beginning of salting, and the rate constant k_2_ is related to the equilibrium concentration of salting. The smaller values of k_1_ and k_2_ indicated that the vacuum impregnation treatment has a higher mass transfer rate at the beginning of the salting and a higher concentration of NaCl when reaching the equilibrium of the salting [20].

The distinct outcomes between vacuum impregnation and control treatments during the brining of grass carp fillets can be attributed to hydrodynamic mechanisms (HDMs) and deformation-relaxation phenomena (DRP). Initially, when fillets are immersed in the brining solution, the internal pore pressure equals the atmospheric pressure. However, under vacuum conditions, the pore pressure exceeds the external pressure. This pressure differential leads to an expansion of the muscle structure, causing dissolved free gasses within the tissues to release into the water. Consequently, the resistance to the penetration of the brining liquid into the muscle is reduced. As the internal and external pressures equalize, the brining liquid rapidly permeates the muscle tissue through its pores. This rapid infiltration accelerates the transfer of salt from the solution into the muscle fibres. The efficiency of this process is heightened by the vacuum impregnation, which not only expedites the salt migration but also enhances the overall salt distribution uniformity compared to the control treatment, where the brining occurs at atmospheric pressure without the pressure differential [21,22,23,24].

### 3.2. Changes in the Water Gain of Grass Carp Fillets, the Content of Released Protein, and the Brining Yield During the Brining

This study investigated the impact of vacuum impregnation on the water content, released protein content, and brining yield of grass carp fillets during the salting process. The yield of fillets may be affected by the exchange of water and salt during the brining [25].

**Figure 2:**
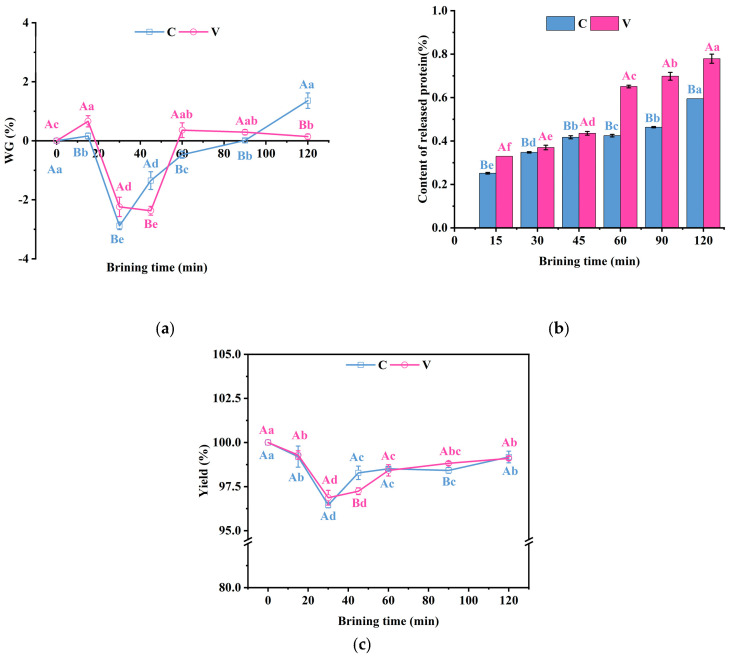
Changes in (**a**) the water gain of grass carp fillets, (**b**) the content of released protein, and (**c**) the brining yield during the brining. V denotes vacuum impregnation and C denotes conventional non-vacuum impregnation at atmospheric pressure. Note: Different capital letters indicate significant differences (*p* < 0.05) between the two treatments; different lowercase letters indicate significant differences (*p* < 0.05) between different brining periods.

Figure 2a illustrates that the net water loss during the curing process is represented by negative values, whereas a net gain is indicated by positive values. Due to the osmotic pressure difference between the low concentration of NaCl solution and the muscle tissue, water may seep out of the muscle cells during the pre-brining period, resulting in muscle water loss and a negative WG. Notably, V group transitioned from water loss to water gain after 60 min, whereas C samples made this transition at 90 min. As salting time increases, the fish muscle binds to the salt ions, causing the myofibrils to swell and absorb water from the salt solution [26,27]. This phenomenon results in an initial negative value of WG in the salt solution, which then turns positive.

The quality attributes of muscle are fundamentally determined by the composition, properties, and structure of its proteins. As depicted in Figure 2b, the protein content within the NaCl solution was quantified during the salting process. The results indicated that the content of proteins released from the grass carp fillets increased significantly (*p* < 0.05) with prolonged brining time, and the protein release in vacuum-treated samples was significantly higher (*p* < 0.05) compared to the atmospheric pressure brining samples. It is well known that a low concentration of NaCl increases the surface charge of protein molecules; this resulted in an increase in the ionic strength of fish muscle facilitating the extraction of muscle proteins [28], and thus the protein content of the NaCl solution increased with the increasing salt content. During this process, myofibrils and the surrounding connective tissue may undergo dissolution. These changes in the content and composition of the released proteins reflect the modifications in the tissue’s ultrastructure, potentially impacting the mass yield and quality characteristics of the fish.

As illustrated in Figure 2c, at 30 min of brining, the yield of both groups dropped to the lowest values of 96.46% and 96.87%, respectively. The yield increased significantly (*p* < 0.05) after 30 min and increased to 99.10% and 99.18% at 120 min. The decrease in yield at the beginning was attributed to water loss due to osmotic pressure, and the subsequent increase in yield was due to cellular uptake of the NaCl solution. In addition to this, the loss of water-soluble proteins as well as other nutrients can also affect yield. Therefore, it is possible to control the duration of brining at 5.8% NaCl solution to obtain a higher mass yield.

### 3.3. Changes in the Microstructure of Grass Carp Fillets During the Brining

**Figure 3:**
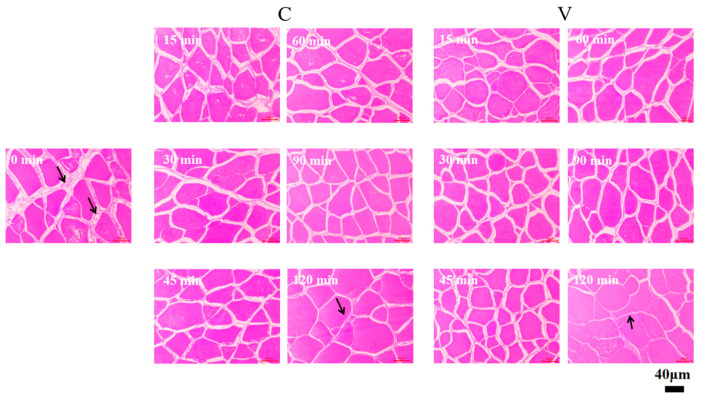
Microstructure of grass carp fillets with prolonged brining time observed by LM. Where V denotes vacuum impregnation and C denotes conventional non-vacuum impregnation at atmospheric pressure. The part indicated by the arrow is the extracellular space.

The microstructural changes in grass carp fillets subjected to both brining treatments were examined using light microscopy (LM). As shown in Figure 3, with longer brining times, the muscle fibre diameters increase and become fuller. Fresh and salted fillets salted for the first 30 min had significant extracellular space (marked by arrows), which is a possible channel for the formation of water loss, giving grass carp fillets a high drip loss and poor water-holding capacity. As the brining time increased, a reduction in extracellular space (marked by arrows) was observed, likely due to myofibrillar swelling resulting from the expansion of the filamentous lattice [29]. The charged groups on the thick and thin filaments normally repel each other due to like charges, contributing to the stability and structure of the muscle fibre. When NaCl is introduced, a large number of chloride ions bind to the myofilaments and the negative charge on the filaments increases, resulting in a stronger electrostatic effect and therefore an increase in electrostatic repulsion [9], which may increase the water-holding space within the tissue. Due to the increase in water, the cell volume of the fish increases and the fibre gaps become small.

### 3.4. Changes in T_2_ Relaxation Characteristics of Grass Carp Fillets During Brining

Low field nuclear magnetic resonance (LF-NMR) relaxometry is a valuable technique for assessing water content and migration in foods. It has been demonstrated that *T*_2_ relaxation is associated with an increase in the overall size of myofibrils and that the transfer of water from the outer space of myofibrils to the expanded intra-myofibrillar matrix is responsible for the changes in *T*_2_ relaxation [30,31]. Briefly, in meat, any change in the *T*_21_ time constant implies a change in water within the myofibrils caused by a change in myofibrils structure. The *T*_22_ time constant represents free water that exists outside myofibrils and reflects changes in membrane properties and to the space of the extra-myofibrillar region. *T*_2b_ represents water which is tightly bound to macromolecules, and it does not change with any mechanical stress in the meat matrix [30]. The transversal relaxation times *T*_2_ and the *T*_2_ population (*P*_2_) of grass carp fillets during brining are shown in Table 2.

Bound water, characterized by its low mobility and tight binding to macromolecules, remains unaffected by mechanical forces [32]. Therefore, the pressure difference generated by a vacuum does not have a significant effect on bound water compared with atmospheric pressure salting in grass carp fillets for 120 min. However, it was found that brining significantly increased (*p* < 0.05) the *T*_2b_ of grass carp fillets. Concurrently, the peak area ratio *P*_2b_ reached its maximum value at 15 min into the salting process and then significantly decreased (*p* < 0.05) thereafter. The *T*_2b_ relaxation characteristics can reflect the availability of protein side chains, which is influenced by the swelling of myofibrils. As the myofilament lattice expands, it increases the surface area within the myofibrils, exposing more macromolecules that can serve as water-binding sites, thus enhancing *P*_2b_. The decrease in hydrophilic groups due to protein denaturation may be responsible for the significant decrease in *P*_2b_. Mcdonnell et al. noted that an increase in *T*_2b_ with increasing NaCl concentration suggests a reduction in the availability of hydrophilic groups for water binding as proteins denature [33].

After brining, the presence of immobile water was increased in salted fish fillets compared to fresh samples, as evidenced by a significant increase in *P*_21_ (*p* < 0.05). The observed significant increase (*p* < 0.05) in *T*_21_ may be caused by the increased distance between thick and thin filaments [34]. Conversely, *P*_22_ was significantly reduced (*p* < 0.05) in both groups of brining, signifying a decrease in the content of free water within the grass carp fillets; this reduction in free water may have contributed to the significant increase in *P*_21_ (*p* < 0.05). Due to the swelling of myofibrils induced by NaCl, the water-holding space inside the myofibrils increases, which allows the free water present on the outside of the myofibrillar dimension to be converted into water inside the myofibrils, increasing the immobile water. The immobile water within the myofibrils was important for the water-holding capacity of the meat products.

### 3.5. Changes in the pH, Cooking Loss and Water-Holding Capacity of Grass Carp Fillets During Brining

Water-holding capacity is a critical quality attribute of fish muscle that reflects its ability to retain water, significantly influencing the texture and taste of the fish as a food product. It is well established that pH is a critical factor in controlling the water-holding capacity of meat products. In live fish, the muscle pH typically ranges from 7.2 to 7.4. However, post-mortem biochemical reactions, including the anaerobic catabolism of glycogen, lead to the production of lactic acid and a subsequent gradual decrease in muscle pH. This decrease, is pivotal as it influences the fish’s ultimate pH and the rate at which it declines.

**Figure 4:**
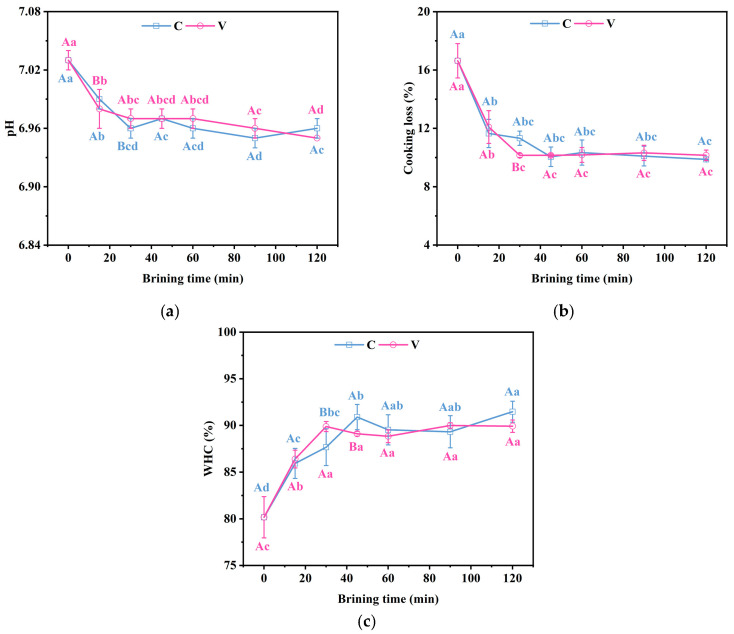
Changes in the pH (**a**), cooking loss (**b**) and water-holding capacity (**c**) of grass carp fillets during brining. V denotes vacuum impregnation and C denotes conventional non-vacuum impregnation at atmospheric pressure. Note: Different capital letters indicate significant differences (*p* < 0.05) between the two treatments; different lowercase letters indicate significant differences (*p* < 0.05) between different brining periods.

In our study, both treatments demonstrated a significant reduction (*p* < 0.05) in pH values throughout the salting, from the initial 7.03 to 6.96 and 6.95, respectively, as shown in Figure 4a. Changes in pH alter the distribution of the net charge of myofibrillar proteins, which determines the electrostatic repulsion between myofibrillar filaments. Following the fish’s demise, the pH dropped and the electrostatic repulsion between myofibrils lessened. This led to myofibril lateral contraction, which weakened the water-holding capacity [35]. Understanding these pH-related changes is essential for optimizing the salting process and maintaining the quality of fish products.

Cooking loss serves as a key indicator of this water-holding capacity. As depicted in Figure 4b, salting significantly reduced (*p* < 0.05) cooking losses in grass carp fillets. Specifically, at 30 min of brining, vacuum-impregnated grass carp fillets reached a minimum cooking loss of 10.15% and atmospheric pressure-impregnated grass carp fillets had a minimum cooking loss of 9.78% at 45 min of brining. After 30 min of salting, there was no significant change in cooking loss between the two treatments (*p* < 0.05). The water-holding capacity of the grass carp fillets increased significantly (*p <* 0.05) following brining (Figure 4c), with all treatments reaching peak water-holding capacity at the end of the brining process.

The decrease in fish pH causes contraction of the myofibrillar lattice, which is connected by proteins to each other and to the cell membrane; if these connections remain intact, then contraction of the myofibrils leads to contraction of the myofibrils, forming gaps between bundles of muscle fibres, as indicated by the arrows in Figure 3. These channels, formed by the gaps between muscle fibres, are crucial as they are considered the primary source of dripping and blowdown losses post-mortem [36]. Myofibril swelling induced by chloride ions during brining increases the water-holding space while simultaneously shrinking the extracellular space, thereby increasing the water-holding capacity.

### 3.6. Changes in the Colour Characteristics of Grass Carp Fillets During Brining

**Figure 5:**
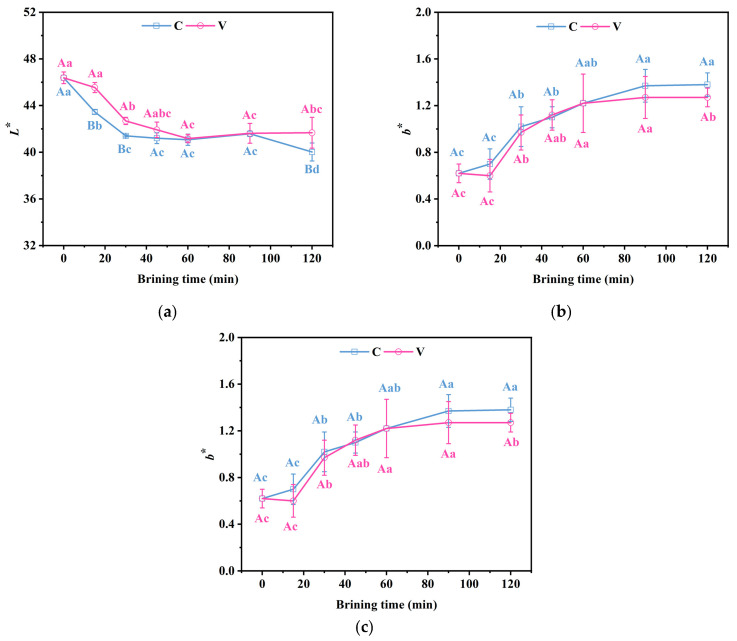
Changes in the (**a**) L* value, (**b**) a* value, and (**c**) b* value of grass carp fillets during brining. V denotes vacuum impregnation and C denotes conventional non-vacuum impregnation at atmospheric pressure. Note: Different capital letters indicate significant differences (*p* < 0.05) between the two treatments; different lowercase letters indicate significant differences (*p* < 0.05) between different brining periods.

Colour characteristics are pivotal factors that influence consumer perceptions of food quality, which significantly affects the acceptability of food products. As depicted in Figure 5a, the L* value, a measure of lightness, of grass carp fillets exhibited a notable decline (*p* < 0.05) following the brining process, decreasing from the initial 46.38 to 40.02 and 41.67. Specifically, the L* value of the V group was significantly higher than that of the C group at 30, 45, and 120 min of brining (*p* < 0.05). This suggests that vacuum impregnation may better preserve the lightness of the fillets. The L* value is correlated with the size of the extracellular space. During brining, the expansion of the myofilament lattice and the reduction in the extracellular space lead to a decrease in light scattering, which manifested as a decrease in L*.

In addition, as shown in Figure 5b,c, the b^*^ values of the two treatments significantly increased (*p* < 0.05) while the a^*^ significantly decreased with the increase in brining time, and there was no significant difference between the a* and b* values of Groups V and C. NaCl promotes the oxidation of meat products, and some compounds produced during lipid oxidation, such as malondialdehyde (MDA) and other aldehydes, may react with the proteins of the food products and other ingredients, resulting in colour changes and increased b* values. Lipid oxidation may accelerate the degradation of pigmented substances such as myoglobin in meat products, and the degradation of these pigments may affect the a* and b* values of meat products. Additionally, the loss of pigment and the increase in water weight can also lead to alterations in the a* and b* values [37].

### 3.7. Changes in Lipid Oxidation of Grass Carp Fillets During Brining

**Figure 6:**
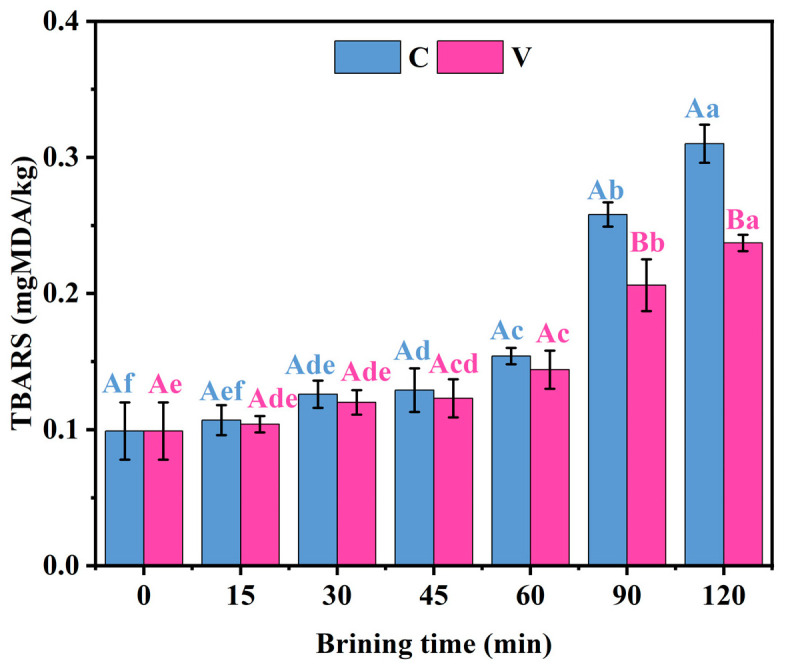
Changes in the TBARS value of grass carp fillets during brining. V denotes vacuum impregnation and C denotes conventional non-vacuum impregnation at atmospheric pressure. Note: Different capital letters indicate significant differences (*p* < 0.05) between the two treatments; different lowercase letters indicate significant differences (*p* < 0.05) between different brining periods.

The TBARS method is a widely utilized technique for assessing lipid oxidation by measuring the relative amount of malondialdehyde (MDA) in a sample. As shown in Figure 6, no significant change (*p* > 0.05) in TBARS values was observed in grass carp fillets at the beginning of brining. However, a significant increase (*p* < 0.05) in TBARS values was detected after 30 min of brining, indicating the production of secondary lipid oxidation products. Qualified fish products should not have a TBARS value exceeding 5 mg/kg [38]. The TBA values of the two groups increased from the initial 0.099 mg MDA/kg to 0.310 and 0.237 mg MDA/kg, respectively. At the 90 min mark, the TBARS values of the C group was significantly higher (*p* < 0.05) compared to those of the V group. This difference may be due to the lower partial pressure of oxygen under vacuum conditions, which can delay the NaCl-promoted lipid oxidation to some extent.

Numerous scholars have proposed that the presence of NaCl in brine can facilitate lipid oxidation in meat products [39]. Several potential mechanisms have been suggested to explain this observation, including the disruption of cell membranes, the promotion of oxidant entry into lipid substrates, and the inhibition of antioxidant enzyme activity [40]. Lipid oxidation products, such as MDA, are known to negatively impact the colour of meat by causing discoloration. Therefore, the samples, which exhibited higher TBARS values, might also have shown a decline in colour quality compared to the V group.

### 3.8. Changes in the Texture Properties of Grass Carp Fillets During the Brining

Fish texture properties are critical sensory indicators for consumers, determining the acceptability of fish and significantly affecting the shelf life of freshwater fish. Therefore, maintaining the textural properties of fish is a key concern in the field of freshwater fish preservation.

**Figure 7:**
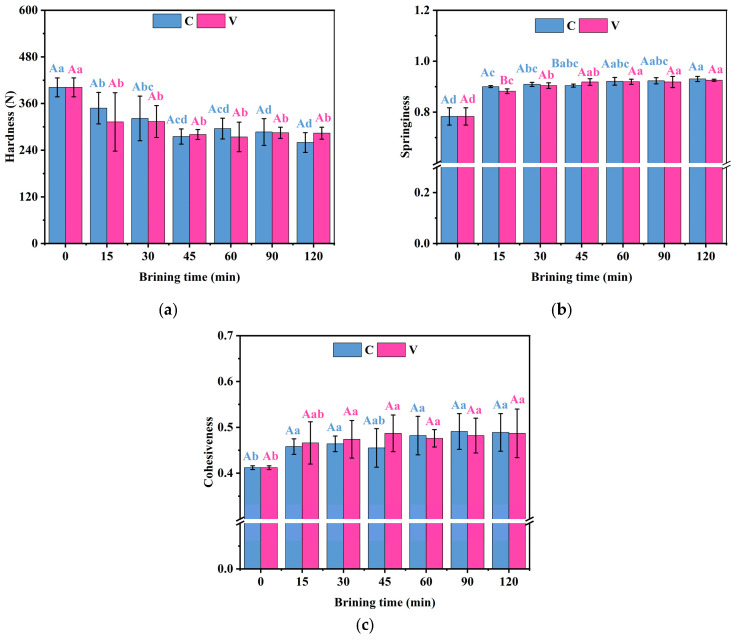
Changes in the (**a**) hardness, (**b**) springiness, and (**c**) cohesiveness of grass carp fillets during brining. V denotes vacuum impregnation and C denotes conventional non-vacuum impregnation at atmospheric pressure. Note: Different capital letters indicate significant differences (*p* < 0.05) between the two treatments; different lowercase letters indicate significant differences (*p* < 0.05) between different brining periods.

As depicted in Figure 7a, the hardness of grass carp fillets significantly declined (*p* < 0.05) during the initial stages of salting for both treatments, followed by a relatively stable condition in the subsequent periods. The decrease in hardness could be attributed to the enhancement of the water-holding capacity during the brining that increased the juiciness and tenderness of the grass carp fillets, while the dissolution of collagen in the connective tissues around the cells also reduced the hardness of the grass carp fillets [41]. Furthermore, the springiness and cohesiveness of the brined grass carp fillets significantly increased (*p* < 0.05). It is speculated that the presence of NaCl promotes the solubilization of salt-soluble proteins and the formation of an ordered protein network structure. This network structure may account for the observed enhancement in springiness and cohesiveness of the grass carp fillets [42]. The combined reduction in hardness, the increase in springiness and cohesiveness, and the high water-holding capacity likely contribute to the distinctive delicate and smooth texture of lightly brined grass carp.

## 4. Conclusions

This study demonstrates that vacuum-assisted brining significantly enhances the efficiency and quality of grass carp fillets compared to traditional atmospheric brining. By applying vacuum conditions (−20 kPa), salt penetration was accelerated, achieving a higher final salt content (2.01%) within 120 min. The Peleg model confirmed superior mass transfer kinetics in vacuum-treated samples, with a lower k_1_ (11.51) indicating faster initial salt diffusion. The microstructure and LF-NMR analysis showed that vacuum salting, reducing extracellular voids, and redistributing free water into fixed forms resulted in a WHC value of 91.47% and 89.92% for fillets, which was superior to that of the fresh fish. Furthermore the hardness of the grass fillets was decreased and the springiness and cohesiveness were increased. In addition, vacuum conditions attenuate lipid oxidation and reduce grass carp fillets’ TBARS values (0.237 mg MDA/kg) compared to atmospheric pressure impregnation (0.310 mg MDA/kg). These findings highlight the potential of vacuum technology in regard to optimizing low-salt salting processes for aquatic products to meet the needs of health-conscious consumers while maintaining product quality. Future research should explore industrial-scale applications and long-term storage effects on fish products to further validate its commercial viability.

## Figures and Tables

**Table 1:** Peleg model constants and coefficients of determination for brined grass carp fillets.

Treatment	k_1_	k_2_	R^2^	RSME
C	15.15 ± 2.59	0.73 ± 0.05	0.9827	0.0397
V	11.51 ± 2.20	0.67 ± 0.06	0.9245	0.0658

**Table 2:** The transversal relaxation times *T*_2_ and the *T*_2_ population (*P*_2_) of grass carp fillets during brining.

Time (min)	Treatment	*T*_2b_	*T*_21_	*T*_22_	*P*_2b_	*P*_21_	*P*_22_
0	C S	1.74 ± 0.07 ^Ab^ 1.74 ± 0.08 ^Ac^	52.34 ± 2.12 ^Ac^ 52.3 ± 2.12 ^Ac^	1430.64 ± 35.28 ^AA^ 1430.64 ± 35.28 ^AA^	2.74 ± 0.15 ^Ab^ 2.74 ± 0.15 ^Abc^	95.60 ± 0.56 ^Ae^ 95.60 ± 0.56 ^Ae^	1.52 ± 0.26 ^AA^ 1.52 ± 0.26 ^AA^
15	C S	5.55 ± 0.38 ^AA^ 5.46 ± 0.83 ^Ab^	60.14 ± 2.44 ^Ab^ 62.95 ± 0.00 ^Ab^	1368.78 ± 57.74 ^AAb^ 1371.88 ± 82.38 ^A Ab^	3.23 ± 0.06 ^A A^ 3.48 ± 0.19 ^A A^	96.45 ± 0.26 ^Ade^ 96.62 ± 0.21 ^Ad^	0.16 ± 0.02 ^Ab^ 0.13 ± 0.05 ^Ab^
30	C S	4.50 ± 0.31 ^AA^ 5.36 ± 1.00 ^Ab^	67.64 ± 0.27 ^AA^ 65.97 ± 2.62 ^AAb^	1130.94 ± 161.73 ^AAbc^ 1380.22 ± 94.77 ^AAb^	2.74 ± 0.24 ^Ab^ 2.81 ± 0.16 ^Ab^	96.73 ± 0.28 ^Ad^ 96.94 ± 0.21 ^Acd^	0.13 ± 0.01 ^Ab^ 0.12 ± 0.00 ^Ab^
45	C S	4.65 ± 0.82 ^AA^ 3.82 ± 0.92 ^Ac^	65.97 ± 2.62 ^AA^ 64.41 ± 2.53 ^A Ab^	1174.53 ± 225.16 ^Abcd^ 1195.93 ± 223.60 ^Abcd^	2.47 ± 0.29 ^Ab^ 2.42 ± 0.32 ^Ac^	97.04 ± 0.41 ^Acd^ 97.40 ± 0.40 ^Ac^	0.09 ± 0.04 ^Ab^ 0.11 ± 0.02 ^Ab^
60	C S	4.73 ± 0.49 ^AA^ 4.82 ± 0.00 ^Abc^	66.00 ± 2.62 ^Ab^ 65.97 ± 2.62 ^AAb^	1190.17 ± 48.24 ^Abcd^ 1268.57 ± 258.17 ^Abcb^	1.90 ± 0.04 ^Ac^ 1.72 ± 0.11 ^Ad^	97.70 ± 0.35 ^Abc^ 98.11 ± 0.13 ^Ab^	0.09 ± 0.01 ^Ab^ 0.10 ± 0.02 ^Ab^
90	C S	4.46 ± 0.93 ^AA^ 5.47 ± 0.94 ^Ab^	65.97 ± 2.62 ^Ab^ 67.48 ± 0.00 ^AA^	1138.57 ± 6.36 ^Acd^ 1131.56 ± 90.47 ^Acd^	1.64 ± 0.12 ^Ac^ 1.66 ± 0.14 ^Ad^	98.01 ± 0.03 ^AAb^ 98.13 ± 0.18 ^Ab^	0.09 ± 0.03 ^Ab^ 0.10 ± 0.04 ^Ab^
120	C S	4.50 ± 0.31 ^AA^ 4.94 ± 0.20 ^AAb^	69.10 ± 2.80 ^AA^ 67.95 ± 0.00 ^AA^	1010.52 ± 39.86 ^Ad^ 1013.96 ± 69.27 ^Ad^	1.85 ± 0.01 ^Ac^ 1.84 ± 0.02 ^Ad^	98.79 ± 0.76 ^AA^ 98.90 ± 0.46 ^A A^	0.11 ± 0.01 ^Ab^ 0.10 ± 0.04 ^Ab^

Note: Different uppercase letters indicate significant differences between the two treatments (*p <* 0.05); different lowercase letters indicate significant differences between the different brining periods (*p <* 0.05).

## Data Availability

The original contributions presented in this study are included in the article. Further inquiries can be directed to the corresponding author.
